# Author Correction: Serological responses and vaccine effectiveness for extended COVID-19 vaccine schedules in England

**DOI:** 10.1038/s41467-022-28393-7

**Published:** 2022-02-02

**Authors:** Gayatri Amirthalingam, Jamie Lopez Bernal, Nick J. Andrews, Heather Whitaker, Charlotte Gower, Julia Stowe, Elise Tessier, Sathyavani Subbarao, Georgina Ireland, Frances Baawuah, Ezra Linley, Lenesha Warrener, Michelle O’Brien, Corinne Whillock, Paul Moss, Shamez N. Ladhani, Kevin E. Brown, Mary E. Ramsay

**Affiliations:** 1 Immunisation and Vaccine Preventable Diseases Division, UK Health Security Agency, London, United Kingdom; 2 Statistics, Modelling and Economics Department, UK Health Security Agency, London, United Kingdom; 3 Brondesbury Medical Centre, Kilburn, London, United Kingdom; 4 Sero-Epidemiolgy Unit, UK Health Security Agency, Manchester, United Kingdom; 5 Virus Reference Department, UK Health Security Agency, London, United Kingdom; 6 Institute of Immunology and Immunotherapy, University of Birmingham, Edgbaston, United Kingdom; 7 Paediatric Infectious Diseases Research Group, St. George’s University of London, London, United Kingdom

**Keywords:** Vaccines, Health policy, Public health, SARS-CoV-2

Correction to: *Nature Communications* 10.1038/s41467-021-27410-5, published online 10 December 2021.

The original version of this Article contained an error in Fig. 4, in which the titles for each section were incorrectly shifted to the left rather than being placed above the corresponding section of the figure. This has been corrected in both the PDF and HTML versions of the Article.

